# Identification of Known and Novel *Arundo donax* L. MicroRNAs and Their Targets Using High-Throughput Sequencing and Degradome Analysis

**DOI:** 10.3390/life12050651

**Published:** 2022-04-27

**Authors:** Silvia Rotunno, Claudia Cocozza, Vitantonio Pantaleo, Paola Leonetti, Loris Bertoldi, Giorgio Valle, Gian Paolo Accotto, Francesco Loreto, Gabriella Stefania Scippa, Laura Miozzi

**Affiliations:** 1 Department of Biosciences and Territory, University of Molise, Contrada Fonte Lappone, 86090 Pesche, Italy; s.rotunno@studenti.unimol.it (S.R.); scippa@unimol.it (G.S.S.); 2 National Research Council of Italy, Institute for Sustainable Plant Protection (CNR-IPSP), Strada delle Cacce 73, 10135 Torino, Italy; gianpaolo.accotto@ipsp.cnr.it (G.P.A.); francesco.loreto@unina.it (F.L.); 3 Department of Agriculture, Food, Environment and Forestry (DAGRI), University of Florence, Via San Bonaventura 13, 50145 Florence, Italy; claudia.cocozza@unifi.it; 4 National Research Council of Italy, Institute for Sustainable Plant Protection (CNR-IPSP), Research Unit of Bari, CNR, 70126 Bari, Italy; vitantonio.pantaleo@cnr.it (V.P.); paola.leonetti@ipsp.cnr.it (P.L.); 5 BMR Genomics S.R.L, Via Redipuglia 22, 35131 Padova, Italy; loris.bertoldi@bmr-genomics.it (L.B.); giorgio.valle@unipd.it (G.V.); 6 Department of Biology, University of Padua, Via Ugo Bassi 58/B, 35131 Padova, Italy; 7 Department of Biology, University of Naples Federico II, Via Cinthia, 26, 80125 Naples, Italy

**Keywords:** giant reed, miRNAs, small RNAs, degradome

## Abstract

MicroRNAs (miRNAs) are a class of non-coding molecules involved in the regulation of a variety of biological processes. They have been identified and characterized in several plant species, but only limited data are available for *Arundo donax* L., one of the most promising bioenergy crops. Here we identified, for the first time, *A. donax* conserved and novel miRNAs together with their targets, through a combined analysis of high-throughput sequencing of small RNAs, transcriptome and degradome data. A total of 134 conserved miRNAs, belonging to 45 families, and 27 novel miRNA candidates were identified, along with the corresponding primary and precursor miRNA sequences. A total of 96 targets, 69 for known miRNAs and 27 for novel miRNA candidates, were also identified by degradome analysis and selected slice sites were validated by 5′-RACE. The identified set of conserved and novel candidate miRNAs, together with their targets, extends our knowledge about miRNAs in monocots and pave the way to further investigations on miRNAs-mediated regulatory processes in *A. donax*, Poaceae and other bioenergy crops.

## 1. Introduction

Improving clean energy production and ensuring universal access to affordable and sustainable energy resources are the main objectives of goal 7 of the seventeen Sustainable Development Goals (SDGs) promoted by the United Nations to end poverty, protect the planet, and ensure peace and prosperity to all people by 2030 (https://www.un.org/sustainabledevelopment/ (accessed on 1 March 2022)). The 2019 Global report from The Food and Land Use Coalition (FOLU) (https://www.foodandlandusecoalition.org/global-report/ (accessed on 1 March 2022)) claims the need to “focus on bioenergy that do not (or only minimally) increase the pressure on land use”, cultivating energy crops that do not compete with food production, forest, or degraded land restoration. In this context, second-generation biofuels, defined as fuels that can be manufactured from various types of non-food biomass, including plant materials and animal waste, represent a promising alternative.

*Arundo donax* L., commonly called giant reed, is a perennial rhizomatous invasive grass belonging to the Poaceae family, often cultivated as an energy crop for second generation biofuels production [1,2]. One of the advantages of *A. donax* cultivation resides in its potential to grow in very low nutrient availability conditions [3] and to be employed for restoration of marginal lands [4] and phytoremediation [5,6]. Indeed, the ability of this plant to cope with different biotic and abiotic stresses and survive in degraded and marginal areas has been widely demonstrated [7,8,9,10,11].

MicroRNAs (miRNAs) are a class of small RNA (sRNA) molecules of 21–24 bp involved in the post-transcriptional regulation of a variety of fundamental biological aspects in eukaryotic organisms, including plants [12]. MiRNAs are transcribed by RNA polymerase II from MIR genes. These sequences, called primary miRNAs (pri-miRNAs), contain a self-complementary region that is processed by Dicer complex; the first cleavage removes the non-complementary part, originating the miRNAs precursor (pre-miRNA), while the second cleavage originates a small double-stranded RNA molecule, known as miRNA/miRNA* duplex, with 2-nt 3′ overhang. AGO1/HSP90 complex binds the duplex and frees the miRNA guide strand, originating a mature RISC complex, that is transported into the cytoplasm, where it can perform its function: cleaving the target transcript or inhibiting its translation [13,14]. Several studies have shown that miRNAs are also strongly involved in the response to abiotic and biotic stress [15] and that plants can activate their physiological responses by expressing some miRNAs that act on stress-related target genes [16].

Despite the economic importance of *A. donax*, its genome has not been sequenced so far, probably due to its complexity [17], and only transcriptomic studies have been performed to investigate its gene content and its molecular responses to the environment [9,10,18,19,20,21,22]. Based on the available transcriptomic data from different tissues (root, bud, culm, and leaf), an in silico prediction of *A. donax* miRNAs was conducted, resulting in the prediction of 141 conserved miRNAs distributed into 14 miRNA families and 462 in silico predicted target transcripts [23]. However, as far as we know, no direct investigation of miRNAs and miRNA targets has been performed so far in Arundo.

**Figure 1:**
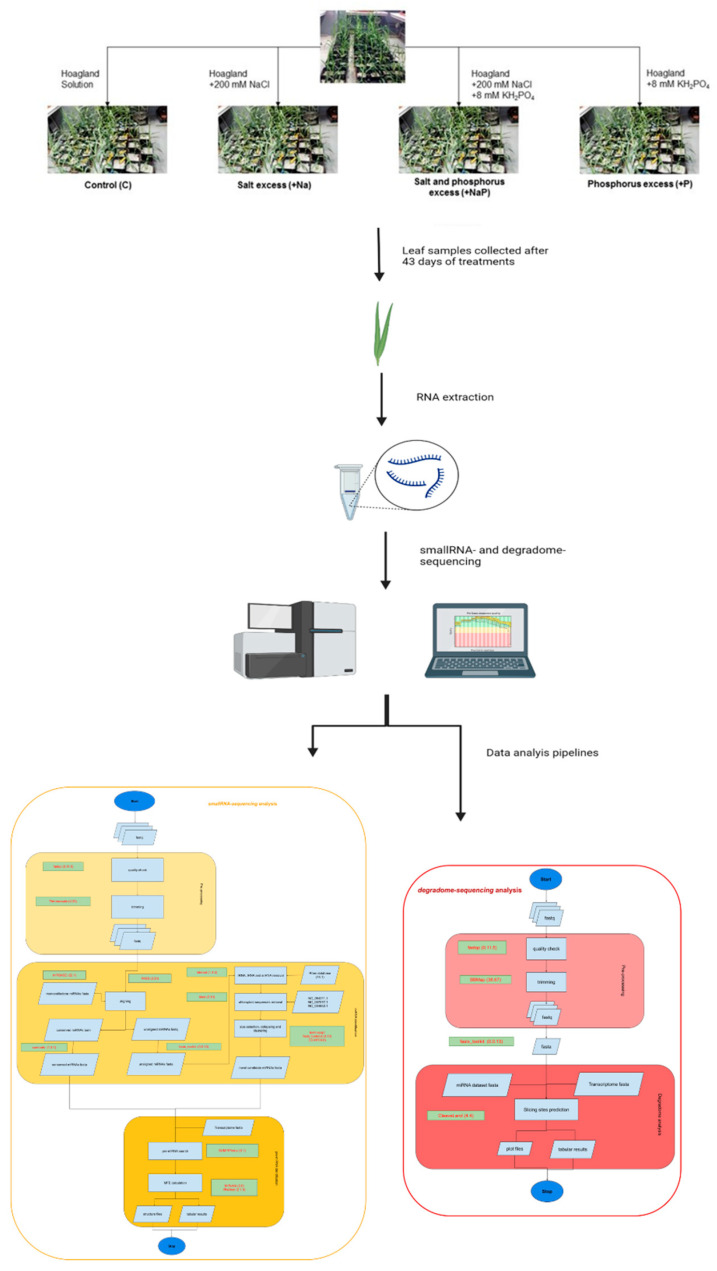
Schematic representation of experimental design and workflow of the performed analyses.

In the present study, we carried out for the first time a high-throughput sequencing approach to identify known miRNAs and putative candidate novel miRNAs of the bioenergy crop *A. donax*, and investigated, by the parallel analysis of RNA ends (degradome analysis) [24], the targets of both known and candidate novel miRNAs in order to explore the miRNA putative functions (Figure 1). Overall, this study advances our knowledge of *A. donax* miRNAs and provides an inventory of miRNAs and related targets useful for further studies, aiming to clarify the typical characteristics of resilience of *A. donax* to biotic and abiotic stress.

## 2. Materials and Methods

### 2.1. Plant Material

*A. donax* plants were propagated from rhizomes collected in Sesto Fiorentino, (43°81′75″ N, 11°18′88″ E) (Italy) and grown in a climatic chamber under controlled environmental conditions for two months before the beginning of the experiment. Four different nutrient solutions were supplied: (1) Hoagland solution (Control, C); (2) Hoagland solution complemented with 8.0 mM KH2PO4 (excess of phosphorus, +P); (3) Hoagland solution complemented with 200 mM NaCl (excess of salt, +Na); and (4) Hoagland solution complemented with both 200 mM NaCl and 8.0 mM KH2PO4 (excess of phosphorus and salt, +NaP). These solutions were supplied twice a week for the entire duration of the experiment (43 days). For each plant, the third fully expanded leaf was collected at the end of the experiment and stored at −80 °C until RNA extraction was performed. Three biological replicates for each treatment were collected. However, considering the complexity and imperfect knowledge of the Arundo genome, in the present study we focused on miRNAs that are common to all stress conditions. Therefore, for sRNAs sequencing, for each treatment, three samples were pooled together, while, for degradome sequencing, a pool from samples of all the treatments was prepared. More experimental details on plant growth conditions, stress treatments and sample collection are reported in the cognate paper [9].

### 2.2. sRNAs and Degradome Sequencing

Total RNA extraction was performed with TRIzol^®^ Reagent (Thermo Fisher Scientific^®^, Wilmington, NC, USA), according to the manufacturer’s instructions. DNA contamination was removed using TURBO DNA-freeTM kit (Thermo Fisher Scientific^®^). RNA concentration was determined by Nanodrop spectrophotometer (Thermo Fisher Scientific^®^). Samples were sent to LCSciences (Houston, TX, USA) for library preparation and sequencing with Illumina technology (single end reads, 50 bp). Raw data have been deposited in the Sequence Read Archive (https://www.ncbi.nlm.nih.gov/sra (accessed on 1 March 2022) with SRA accession PRJNA774159.

### 2.3. Data Analysis

Quality reads assessment of sequencing data was carried out with FastQC (http://www.bioinformatics.babraham.ac.uk/projects/fastqc/ (accessed on 1 March 2022) v. 0.11.5). In order to identify known miRNAs, after adapter removal with Trimmomatic (v 0.39; http://www.usadellab.org/cms/?page=trimmomatic (accessed on 1 March 2022)) [25], clean reads were aligned to mature monocotyledons miRNAs (downloaded from miRBase v 22.1; https://www.mirbase.org/ (accessed on 1 March 2022)) with PASS software (v. 2.30; http://pass.cribi.unipd.it (accessed on 1 March 2022)) [26] using the following parameters: read trimming with a quality threshold of 15 in a window of 6 nt, local alignment with ungapped seed structure, 89.9% as identity percentage (i.e., 2 mismatches) and 18 nt as minimal alignment size. Identical mapped reads were collapsed with the tool fastx_collapser belonging to the FASTX toolkit (v. 0.0.13; http://hannonlab.cshl.edu/fastx_toolkit/ (accessed on 1 March 2022)) and, if present in at least three libraries (i.e., three different treatments) with a minimum of 5 reads, were renamed according to the corresponding miRNAs. Primary miRNA sequences (pri-miRNA) were searched in the *A. donax* transcriptome [9] using the Perl script SumirFind.pl (v. 1.1; https://github.com/MiqueiasFernandes/bioinformatics/blob/master/SUmirFind_sRNA.pl (accessed on 1 March 2022)) [27,28] allowing two mismatches. MIReNA tool (v. 2.0; http://www.lcqb.upmc.fr/mirena/index.html (accessed on 1 March 2022)) [29] was then used for precursors (pre-miRNAs) sequences identification and validation and for calculation of Minimum Folding Energy (MFE) and Minimal Folding Energy Index (MFEI). MFE values estimated the stability of the stem-loop structure of the miRNA precursor whereas MFEI values were used to distinguish pre-miRNA sequences from other coding or non-coding RNAs. Secondary structure design was carried out with RNAfold [30]. miRNAs* sequences were defined as the strand with the lower frequency between the two complementary strands.

For novel miRNA prediction, unaligned reads were scanned with Infernal (v. 1.1.2) in order to remove rRNA, tRNA and snoRNA, and then blasted against the complete chloroplast genomes of three *Arundo* species, i.e., *A. formosana* (NC_054211.1), *A. donax* (NC_037077.1), and *A. plinii* (NC_034652.1) available in the NCBI RefSeq database, to remove chloroplastic sequences. Reads were then size selected (less than 24 nt long), collapsed with fastx_collapser (FASTX toolkit, v. 0.0.13), and clustered using CD-HIT software (v. 4.6.6), using a word size of 5 and 85% identity in order to reduce redundancy. After checking the presence in at least three different libraries (i.e., three different treatments), reads were selected for further analyses as hypothetical novel miRNAs. Pri-miRNA, pre-miRNA, and miRNA* sequences were identified as described above for known miRNAs. Secondary structures of pre-miRNAs were obtained using the RNAfold web server (http://rna.tbi.univie.ac.at//cgi-bin/RNAWebSuite/RNAfold.cgi (accessed on 1 March 2022)).

In the case of degradome-sequencing data, adapters and low-quality reads trimming was performed with BBMap (v. 38.67; https://jgi.doe.gov/data-and-tools/software-tools/bbtools/). Clean reads were then used to identify miRNA sliced target sites using the CleaveLand 4 pipeline (v. 4.4; [31]). According to the relative abundance of the mapping reads, sliced target sites were distributed into five categories, defined as follows. Category 0: more than one read mapping at the cleavage position, equal to the maximum on the transcript, when there is just one position at the maximum value; category 1: more than one read mapping at the cleavage position, equal to the maximum on the transcript, when there is more than one position at maximum value; category 2: more than one read mapping at the cleavage position, above the average depth, but not the maximum on the transcript; category 3: more than one read mapping at the cleavage position, but below or equal to the average depth of coverage on the transcript; category 4: just one read mapping at the cleavage position. GO annotation of target genes was carried out with Blast2GO tool [32].

### 2.4. 5′ RACE for Cleavage Site Identification of miRNA Targets

Poly(A) RNA fraction from total RNA was collected using the MicroPoly(A) Purist™ kit (Thermo Fisher Scientific^®^). Generation of cDNAs of poly-adenylated 5′ remnants ligated to 5′ adapter was carried out according to the protocol reported in [24]. PCR was performed using 5′ adapter primer and 3′specific primer (denoted by the target id in Table S1). PCR conditions were initial denaturation at 98 °C for 30″ followed by 34 cycles of denaturation at 98 °C for 20″, annealing at 60 °C for 30″ and elongation at 72 °C for 15″; final elongation was performed at 72 °C for 3′. PCR products were resolved in 1X TRIS-Borate buffer, 8% acrylamide gel, eluted in sterilized water at 4 °C, overnight and precipitated adding 2.5 *v/v* of absolute ethanol and 1/10 of 2 M NaCl. Purified PCR products were subjected to a second round of amplification with the same pairs of primers, purified using the MinElute^®^ PCR Purification kit (Qiagen, Hilden, Germany), and cloned into the pGEM^®^-T Easy Vector System I (Promega, Madison, WI, USA). Clones were sent to BMR Genomics (Padova, Italy) for Sanger sequencing.

## 3. Results

### 3.1. sRNA Profiles of A. donax

To identify miRNAs from A. donax, sRNA libraries were generated from total RNA extracted for each of the following treatments: (1) control plants (C); (2) excess of phosphorus (+P); (3) excess of sodium (+Na); (4) excess of sodium and phosphorus (+NaP). Libraries were then sequenced with the high-throughput Illumina technology. Since the aim of the work was to obtain a catalogue of known and novel miRNAs and related targets in A. donax, a species with a complex and imperfectly known genome, we considered sRNAs libraries as biological replicates and focused on miRNAs common to at least three libraries. After trimming of adapters, removing of low-quality sequences, rRNA, tRNA and snoRNA, and selection of reads shorter than 29 nt, a total of clean reads ranging between 2,545,750 (+Na) and 4,096,100 (+P) were retained for further analyses (Table S2).

**Figure 2:**
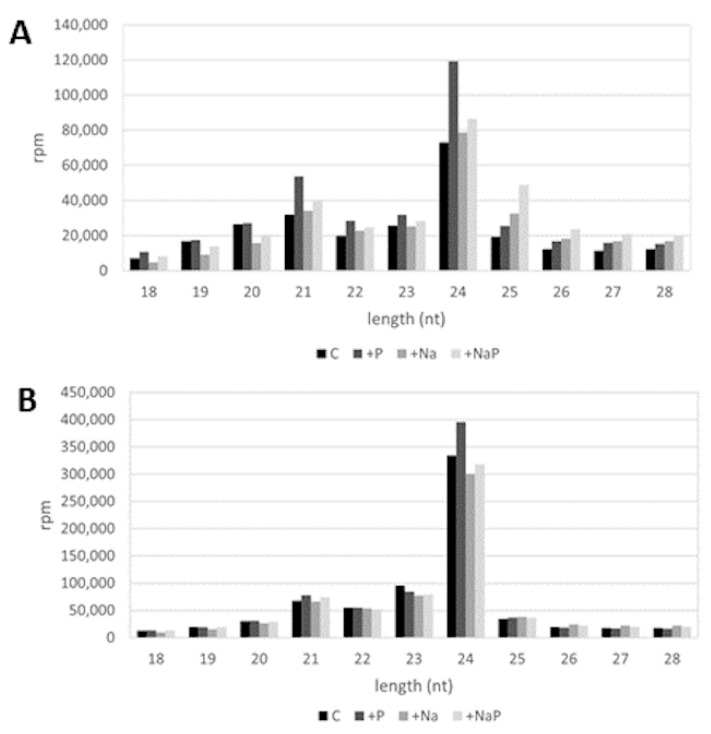
Length distribution of (**A**) redundant and (**B**) non-redundant reads in C, +P, +Na, +NaP libraries; rpm: reads per million.

In all the libraries, the majority of sRNA reads ranged between 20- and 24-nt in length (C, 69.1%; +P, 71.9%; +Na, 64.1%; +NaP, 59.6%); for all treatments, the most abundant sRNAs were 24-nt in length, followed by the 21-nt sRNAs (Figure 2). As expected, being miRNAs mostly 21-nt long, in all treatments 21-nt sRNAs showed a doubled level of redundancy compared to 24 nt-sRNAs (estimated by the average complexity index of 0.30 and 0.65, respectively).

### 3.2. Identification of Known miRNAs in A. donax

**Figure 3:**
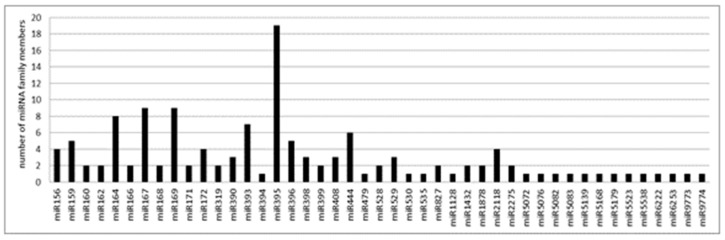
Number of miRNA members for each conserved miRNA family identified in *A. donax* L.

To limit the number of false positives and improve the accuracy of the annotation, we annotated as known miRNAs only those present in at least three libraries with a minimum of 5 reads per library. According to these criteria, a total of 134 known miRNAs, belonging to 45 miRNA families, were identified. Among them, the family of miR395 was the most represented with 19 members, followed by the family of miR167 and miR169, both with 9 members, and the one of miR164 represented by 8 members. By contrast, several miRNA families were represented by 2 or 1 member (Figure 3 and Table S3). According to the last release of the miRbase database (v22.1), among the 45 miRNA families, 29 were conserved in both monocot and dicot clades, 15 were identified so far only in the Poaceae grass family and 1 in both Poaceae and Bromeliaceae (both belonging to the order of Poales).

**Figure 4:**
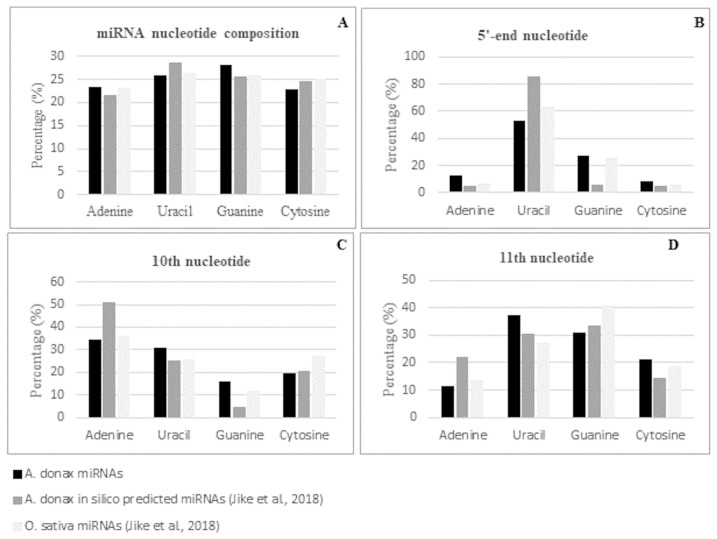
Nucleotide composition (**A**) and 5′ (**B**), 10th (**C**), and 11th (**D**) -nucleotide distribution of *A. donax* L. mature known miRNAs.

As expected, the majority of conserved miRNAs were of 21-nt, followed by those of 22-nt, i.e., 67% and 20%, respectively. The overall nucleotide composition of the mature miRNA sequences was 25.89% of uracil, 23.21% of adenine, 28.17% of guanine, and 22.72% of cytosine with a GC content of 50.89% (Table S4). These values are in line with the base composition reported previously for the subset of Arundo conserved miRNAs identified by an in silico analysis of transcriptomic data and for that of the mature miRNAs in Oryza sativa [23] (Figure 4A). Position specific nucleotide sequence analysis of the identified conserved miRNAs revealed that uracil was the most represented nucleotide at the 5′ end, with a percentage of 52.99% (Figure S1A). This value is in line with the percentage observed in rice (62.71%) and is lower than the value of 85.11% observed in the miRNA subset previously identified by [23] (Figure 4B). Focusing on position 10 and 11, considered as the two principal common cleavage sites, we found that, in agreement with what was observed for rice miRNAs [23], adenine was the most represented nucleotide at position 10 (34.33%), while uracil and guanine were the two most represented nucleotides at position 11, with a percentage of 37.31% and 30.60%, respectively (Figure 4C,D and Table S4). The differences observed in respect to the nucleotide distribution found in mature miRNAs by [23] could reside in the fact that, due to the in silico transcriptomic-based approach used, only a subset of highly conserved miRNA families was detected.

MIReNA analysis allowed us to predict the pre-miRNA sequences of 29 known miRNAs, with a length ranging from 60- and 217-nt and an average length of 121-nt. The reliability of the pre-miRNA’s prediction was supported by the highly negative MFE values, ranging from −122.3 kcal/mol to −26.0 kcal/mol. Moreover, the pre-miRNAs MFEI values, ranging between −1.48 kcal/mol and −0.85 kcal/mol, clearly indicated that they do not belong to other classes of non-coding RNAs such as tRNAs (MFEI = −0.64 kcal/mol), rRNAs (MFEI = −0.59 kcal/mol), and mRNAs (MFEI = 0.62–0.66 kcal/mol) [33,34].

Comparing the number of identified miRNA loci with that reported in miRBase (v22.1) for Oryza sativa and by [23] in the Arundo in silico annotation, we confirmed that the family of miR169 has the highest number of loci both in *O. sativa* and *A. donax* (Table 1). In line with our results, miR169 has been identified so far in more than 40 species [35], and it is often the miRNA family with the largest number of loci [36]. The miR395 family, for which we identified 8 loci, was the second most represented miRNA family in *A. donax*.

**Figure 5:**
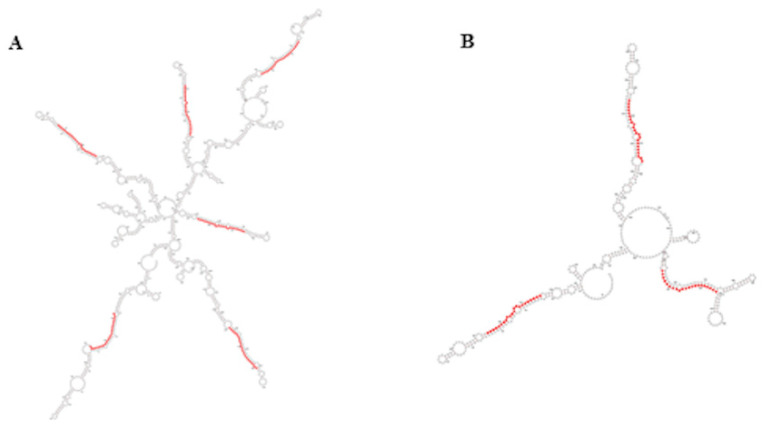
Secondary structure of miR395 clusters belonging to transcripts TR3796 (**A**) and TR16038 (**B**); sequences of miR395 are highlighted in red. Secondary structures were obtained by RNAfold webserver.

In plants, the miR395 family is one of those with more members and, in rice and wheat, it has been found to be organized in compact clusters originated by reiterated duplication events and transcribed as one single polycistronic transcript [37,38]. The compact polycistronic structure of this miRNA family is conserved in *A. donax*, where several miR395 family members originate from the same 2 transcripts (Figure 5A,B), thus suggesting a similar strategy of expression.

The abundance of known miRNA reads was estimated as transcript per million (TPM). The obtained TPM values widely varied among the miRNA families identified. Members of the conserved miRNA families miR167, miR168, miR169, and miR396 as well as members of monocots-specific miRNA families miR2275, miR5072, and miR5168 were the most highly expressed miRNAs (TPM > 10,000). Among those with a TPM ranging between 10,000 and 1000, we found several miRNAs families conserved in both monocots and dicots (miR156, miR159, miR160, miR162, miR164, miR168, miR169, miR171, miR172, miR393, miR394, miR827) and few members of monocots-specific miRNA families (miR444, miR9773, miR9774) (Table S3).

### 3.3. Identification of Novel Candidate miRNAs in A. donax

Based on the recently revised criteria for the annotation of plant miRNAs [39], 27 candidate novel miRNAs (miRCs) were identified along with their star sequences (Table S5). As for known miRNAs, miRCs were considered only if sequences were present in at least three libraries with a minimum of 5 reads per library.

As expected, *A. donax* miRCs were mostly of 21 nt (Table S5). The overall nucleotide composition of the miRC sequences was 25.69% of uracil, 25.87% of adenine, 23.96% of guanine, and 24.48% of cytosine with a GC content of 48.44% (Table S4). Position specific nucleotide sequence analysis highlighted that adenine was the most represented nucleotide at the 5′ end, with a percentage of 40.74%; moreover, guanine and uracil were the most represented nucleotides at position 10 and 11, with a percentage of 40.74% and 29.63%, respectively (Figure S1B and Table S4).

**Figure 6:**
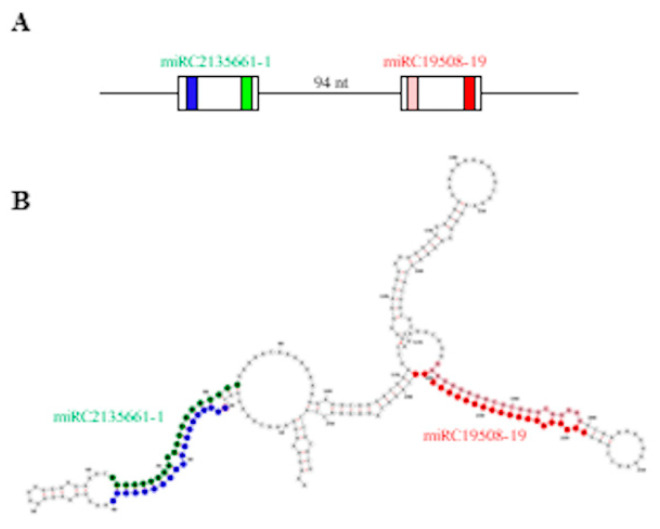
Cluster of novel miRNA candidates miRC2135661-1 and miRC19508-19. (**A**) Schematic representation of the cluster; white boxes represent the predicted pre-miRNAs. (**B**) Secondary structure of transcript TR25104, hosting the cluster. For miRC2135661-1, green and blue represent the mature miRC and miRC*, respectively. For miRC19508-19, red and pink represent the mature miRC and miRC*, respectively. Secondary structure was obtained by RNAfold webserver.

Pre-miRNA sequences predicted by MIReNA analysis ranged from 60 to 252 nt in length, with an average length of 114 nt. Similarly to the pre-miRNAs of known miRNAs, the negative MFE (ranging from −124.5 kcal/mol to −19.9 kcal/mol) and MFEI values (ranging from −1.90 to −0.94) supported the reliability of predictions. The existence of a cluster originating from the same transcript and containing the pre-miRNA sequences of two different novel miRCs, i.e., miRC2135661-1 and MIRC19508-19, spaced 94-bp apart (Figure 6A,B) prompted us to hypothesize the coordinated expression of these miRCs and their involvement in the regulation of the same biological process. Indeed, even if no targets were identified by the degradome analysis, the in silico target prediction performed by psRNAtarget [40] identified two methyltransferase coding transcripts (TR5979 and TR20503, respectively) among the most reliable targets of these miRCs (Table S6).

As already reported in previous studies [41,42,43], the reads abundance of newly identified miRCs, estimated as transcript per million (TPM), were generally lower in respect to the conserved miRNAs, a characteristic possibly explained by the recent evolutionary origin of species-specific novel miRNAs [44]. With a TPM higher than 1000, miRC10722-29 and miRC12006-27 were the most represented in all the considered libraries; seven miRCs (miRC45695-9, miRC22398-17, miRC76001-6, miRC1648-141, miRC71512-6, miRC19508-19, miRC2508-96) had a TPM value higher than 100 in at least two libraries.

### 3.4. miRNAs and miRCs Targets Identification and Functional Characterization

*A. donax* miRNAs and miRCs targets were identified by high-throughput degradome sequencing [24]. A total of 5,897,713 raw reads, corresponding to 796,012 unique sequences, were obtained. After adapters and low-quality reads trimming, a total of 1,557,573 clean reads, representing 464,300 unique reads, were retained. To identify miRNA targets, these reads were analyzed by CleaveLand 4 pipeline (v. 4.4), using the *A. donax* transcriptome previously assembled from the same samples for mapping [9]. It was found that 67% of degradome reads mapped to the *A. donax* transcriptome. We identified 69 targets for 49 conserved miRNAs belonging to 26 miRNA families, including 8 Poaceae-specific families. In the case of miRCs, 27 targets for 9 miRCs were identified. According to the relative abundance of the mapping reads, sliced target sites were categorized into five categories, from 0 to 4 (Figure S2; see Material and Method for category description). The most relevant targets (category from 0 to 3) are reported in Table 2 while the complete list of targets is reported in Tables S3 and S5.

The GO functional analysis of targets of both miRNAs and miRCs highlighted their involvement in different biological processes mainly related to regulation, response to stimuli, primary metabolism, and cellular processes (Figure S3). The presence of several targets related to regulation and response to stimuli is in line with the regulatory nature of miRNAs and with the used experimental design.

### 3.5. Validation of miRNAs and miRCs Cleavage Sites

To verify the cleavage sites identified by the degradome analysis and to confirm their nucleotide position, 5′ RACE experiments were conducted on selected targets, i.e., an oxygen-evolving enhancer chloroplastic protein coding transcript (TR10651) targeted by miRC174433-3 and a 32 kDa dirigent-like coding transcript (TR4471) targeted by miR156 d-3p (Table 2).

**Figure 7:**
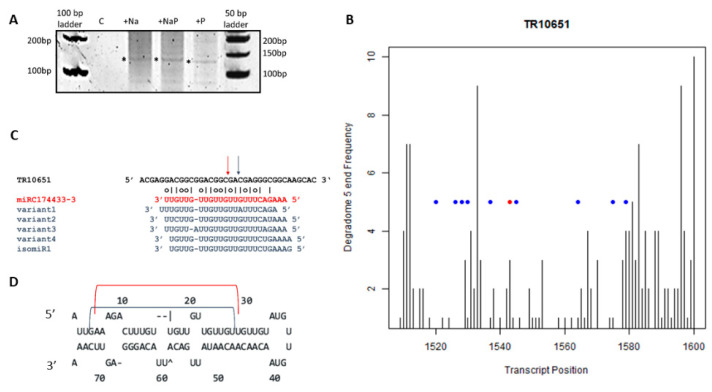
5′ RACE on transcript TR10651 coding for an oxygen-evolving enhancer chloroplastic protein targeted by miRC174433-3. (**A**) Fragment of 120 bp amplified by 5′ RACE in all treatments, except the control treatment, (*) indicates the eluted band; (**B**) CleaveLand plot for TR10651 in the range of 1500–1600 nt, red dot indicates the slicing site found by CleaveLand, blue dots indicate sites derived from Sanger sequencing of clones obtained by 5′ RACE; (**C**) Schematic representation of miRC174433-3 cleavage site. Red arrow indicates the cleavage site confirmed by CleaveLand; blue arrow indicates the cleavage sites found by 5′ RACE analysis. Possible isomiRs are reported in blue; (**D**) pre-miRNA hairpin, the red bracket represents the position of miRC174433-3 and the blue bracket represents the position of miRC174433-3 variants.

By 5′ RACE of the transcript TR10651, we could amplify a specific fragment of ca. 120 bp in all treatments except the control (Figure 7A). The Sanger sequencing of 13 independent clones confirmed 5′ remnants found in the degradome analysis in the range of positions from 1520 to 1580 of TR10651 (Figure 7B, blue dots), including two cases (out of 13) of cleavage sites located two nucleotides downstream the CleaveLand predicted cleavage site (Figure 7B, blue dot downstream red dot). Possible explanations include the presence of other endonucleases cleavage sites or accumulation of particularly stable decay intermediates [24]. The cleavages 2-nt downstream of the red dot (Figure 7B) could be explained by the presence of miRC174433-3 isoforms (denotes as isomiRs, i.e., miRNAs deriving from the same precursor), that have sequence offset at their 5′ ends (Figure 7C,D) or other not yet identified pre-miRNA variants. A similar situation has been found in the case of osa-miR171 [45].

According to the degradome analysis, the miR156d-3p was identified to guide the cleavage of the transcript TR4471, coding for a 32kDa dirigent-like protein, at position 1749. The results obtained with 5′ RACE showed a double fragment in the +NaP sample. Examining the CleaveLand pipeline steps, we found the presence in the degradome library of 5′ remnants confirming the existence of a double cleavage site for miR156d-3p, located at position 1749 and 1830 and compatible with 5′ RACE results; however, due to stringent set up, only the first one was reported in the final CleaveLand output (Figure S4).

## 4. Discussion

With the development of high-throughput sequencing, miRNAs have been identified from various plant species. The identification and characterization of miRNAs may be particularly relevant for *A. donax*, having a surprising resilient character to adverse environmental conditions either biotic or abiotic, resulting in high yields and low agronomic input requirements [46,47]. However, probably due to the complexity of its not yet fully sequenced genome, only an in silico miRNA prediction, based on transcriptomic data, has been performed so far [48].

In the present study, small RNAs from *A. donax* plants were sequenced and known as well as candidate novel miRNAs were identified. The obtained sRNA profiles were consistent with the typical ones reported for rice, wheat, and other monocots [43,49,50,51], where the 24-nt sRNAs are the most abundant and heterogeneous class of small non-coding RNAs in the sRNAs libraries, while the 21-nt sRNAs, being mostly miRNA sequences, show a low level of redundancy. Consistently with the general characteristic of miRNAs [39] and with previous reports on *A. donax* [48] and other plants [43,52], we observed a miRNA length distribution with a prevalence of 21-nt sequences followed by those of 20-nt in length. Furthermore, the observed 5′ enrichment of uracil as well as the overall nucleotide composition of the mature miRNA sequences (Table S4) are comparable with the values reported previously for in silico predicted *A. donax* mature miRNAs and for *O. sativa* [48] and are in line with the known preference of the AGO1 protein for harboring miRNAs with a 5’ terminal uracil [53]. Finally, the reliability of predictions is supported by the length of precursors beneath 300-nt [53] as well as by the negative MFE and MFEI values obtained for both miRNAs and miRCs [33,34]. Comparing the number of miRNA loci with those found in the rice genome, we observed that major and most conserved miRNA families are widely represented also in *A. donax*. Among these families, it is worth mentioning miR395 whose clustered nature is strongly conserved. The presence of clusters of miRNA genes transcribed in large polycistronic primary transcripts is frequent in animals but only few conserved cases have been described so far in plants, suggesting that the clustering may be critical for the coordinate regulation of these specific miRNAs [54].

To identify the *A. donax* miRNA targets, a degradome high-throughput sequencing approach was performed and 5′ RACE experiments were conducted to confirm the 5′ cleavage remnants for specific cases. This approach allowed us to confirm the functionality of conserved miRNAs and to extend our knowledge as well. For instance, we could identify the miRC174433-3 and its isomiRs, possibly originated from the pre-miRC174433-3 or from other not yet identified pre-miRNA genes. The length of miRC174433-3 and its variants ranged from 23- to 24-nt. Previous studies have shown that precursors representing most conserved miRNA families are also independently processed by DCL3 (in addition or in alternative to the canonical DCL1 cleavage) to generate miRNAs that are 23–25-nt in length, called long miRNAs [55]. This evidence has been confirmed in several plants [56,57]. Moreover, the accumulation of miRNAs longer than 21-nt in A. thaliana was shown to be inversely correlated with the level of miRNA conservation [55]. Such evidence prompted us to hypothesize the existence of additional isomiR genes, besides the novel candidate miRNA gene pre-miRC174433-3 (Figure 7C,D) and highlights the importance of specific analyses of the transcriptome assembly in order to unravel more isomiRs. Interestingly, pre-miRC174433-3 shows a structure with a “minimal” loop (Figure 7D). Similarly, osa-miR531, sbi-miR5383, and bdi-miR1135, deriving from precursors with minimal loops, are 23- to 24-nt long [58,59,60]. The function of 23–24-nt miRNAs has not been determined yet; however, they look to be dependent on the organ-specific expression of DCL3 and the hierarchical action of other DCLs, upon biotic or abiotic stresses [56].

Consistent with the nature of samples used for the analysis, among the conserved miRNAs families we identified, several are known to be responsive to stress, i.e., miR156, miR160, miR164, miR167, miR169, miR396, miR399, miR528, and miR827 [16]. The family of miR169, the most abundant in terms of loci also in *A. donax*, has been reported to be involved in stress tolerance and responses to high salinity, and to abiotic stresses in general [14,16]. By the degradome analysis, we identified two targets, respectively of miR169a and miR169c-5p, coding for an mRNA cleavage and polyadenylation factor (CLP1) homolog and a Vascular Plant One-Zinc-Finger (VOZ) 1-like transcription factor. The first one is involved in the 3′-end cleavage and polyadenylation of eukaryotic mRNAs, a critical step of gene expression, that has also been recently related to the regulation of stress responses [61]. Vascular Plant One-Zinc-Finger (VOZ) Transcription Factors are involved in regulating numerous biological processes such as floral induction and development, defense against pathogens, and responses to multiple types of abiotic stress [62,63]. In particular, VOZ transcription factors positively affect salt stress tolerance through the regulation of many stress-responsive genes [64,65] and may be considered a promising target for the generation of salt-tolerant crops.

Among the conserved miRNAs, we identified miR528-5p, a miRNA previously established as a multistress regulator, that has been shown to positively regulate rice salt tolerance by down-regulating a gene encoding L-ascorbate oxidase (AO), thereby bolstering up the AO-mediated abscisic acid synthesis and ROS scavenging [66]. According to our data, miR528-5p targets an L-ascorbate oxidase coding transcript also in *A. donax*, thus suggesting that this regulatory mechanism is conserved among species.

Among the miRCs, it is worth mentioning miRC35861-11 and miRC37052-11. The first one was found to target a geranylgeranyl pyrophosphate synthase coding transcript, an enzyme that in sweet potato is involved in the biosynthesis of carotenoids and is likely associated with tolerance to osmotic stress [67]. Since in *A. donax*, the excess of salt does enhance the biosynthesis of carotenoids [9], further analyses would be useful to investigate the possible involvement of miRC35861-11 in the *A. donax* response to osmotic stress through the regulation of the carotenoid biosynthesis pathway. The miRC37052-11 targets a PAD4 lipase-like coding transcript. In *Arabidopsis* this enzyme is important for the activation of defense responses dependent from salicylic acid, a phytohormone largely recognized as a key player in plant abiotic stress-tolerance [68]. Therefore, miRC37052-11 is a good candidate to be evaluated for its possible involvement in a salicylic acid-mediated response of *A. donax* to stress conditions.

## 5. Conclusions

To conclude, despite the complexity of the *A. donax* genome and the lack of its complete genomic sequence, we identified several known miRNAs and associated pre-miRNAs, expanding the list of species hosting conserved miRNAs in their genome. As mining of species-specific miRNAs has still not been carried out in *A. donax*, we proceeded with a high-throughput sequencing-based annotation of novel candidate miRNAs and their targets and identified new species-specific miRNAs and associated targets. Far from being exhaustive, our results constitute a first step towards a more extensive catalogue of *A. donax* microRNAs, mandatory for attaining a more complete understanding of the microRNA regulatory role in this species, and point out the need to improve the genome and transcriptome data resolution of this economically important crop.

## Figures and Tables

**Table 1:** Comparison between the number of miRNA loci found in *A. donax* in this work with those found in *O. sativa* and previously in silico predicted in *A. donax* by [23].

miRNA Family	*A. donax*	*O. sativa*	*A. donax* [23]
miR156	3	12	8
miR159	2	6	-
miR160	1	6	4
miR162	1	2	-
miR164	2	6	-
miR167	3	10	5
miR169	10	18	16
miR172	1	4	3
miR319	2	2	2
miR393	3	2	4
miR395	8	24	-
miR396	1	8	2
miR398	1	4	-
miR444	5	6	12
miR528	2	1	-
miR6222	1	-	-
miR9774	1	-	-

**Table 2:** List of miRNAs and miRCs targets with the corresponding CleaveLand category and *p*-value (only targets with CleaveLand category below or equal to 3 are reported); targets selected for confirmation by 5′ RACE are in bold; n/a, not annotated.

	Name	Target Id	Target Annotation	Category	*p*-Value
**Known miRNAs**	miR1128	TR24300	Senescence-associated protein (partial)	2	0.082
	**miR156d-3p**	**TR4471**	**32 kDa dirigent-like protein**	**2**	**0.037**
	miR156j-5p.2	TR14474	Hypothetical protein	0	0.007
	miR159a-3p	TR5918	n/a	3	0.055
	miR159b	TR24324	n/a	3	0.058
	miR159b	TR30640	n/a	0	0.059
	miR159e	TR11309	BEL1-like homeodomain 4 containing protein	2	0.025
	miR159e	TR4218	Uncharacterized protein	3	0.046
	miR159e	TR6528	Ankyrin repeat domain-containing 13B	0	0.066
	miR167c-5p	TR29148	ATP synthase delta chloroplastic protein	2	0.005
	miR169c-3p	TR24300	Senescence-associated protein (partial)	2	0.051
	miR172b	TR25857	Hypothetical protein	3	0.086
	miR172d-5p	TR29998	Pentatricopeptide repeat-containing protein	3	0.036
	miR319a	TR1733	Calcineurin B 1	3	0.034
	miR319b-3p	TR24557	n/a	0	0.026
	miR396b	TR20898	Chlorophyll a-b binding chloroplastic-like protein	2	0.062
	miR528-3p	TR1686	n/a	3	0.266
	miR528-3p	TR8922	Peptidyl-prolyl cis-trans isomerase-like 3 protein	0	0.313
	miR529a	TR20898	n/a	3	0.163
	miR6253	TR13145	Serine carboxypeptidase II-3	3	0.066
	miR9774	TR9615	F-box SKIP22	3	0.022
**Candidate miRNAs**	**miRC174433-3**	**TR10651**	**Oxygen-evolving enhancer chloroplastic protein**	**3**	**0.553**
	miRC174433-3	TR27080	Gamma-glutamyl peptidase 5-like	2	0.063
	miRC174433-3	TR8896	Hypothetical protein	0	0.141
	miRC366946-2	TR28663	Cytochrome P450	2	0.005
	miRC71512-6	TR24307	B-box zinc finger 24	2	0.064
	miRC808846-2	TR19738	n/a	3	0.083

## Data Availability

The data that support the findings of this study are included in the manuscript/supplementary files. Raw sequencing data are openly available in the Sequence Read Archive (https://www.ncbi.nlm.nih.gov/sra (accessed on 1 March 2022)) at SRA accession PRJNA774159.

## Supplementary Materials

The following supporting information can be downloaded at: https://www.mdpi.com/article/10.3390/life12050651/s1. Table S1. Primer sequences and melting temperature used for PCRs in 5′ RACE. Table S2. Reads statistics of small RNAs high-throughput sequencing. Table S3. *A. donax* conserved miRNAs and their targets. Table S4. Base composition of *A. donax* conserved miRNAs and comparison with Jike et al. (2018). Table S5. *A. donax* miRCs and their targets. Figure S1. Nucleotide composition of miRNAs and miRCs found in *A. donax*. Figure S2. Summary of cleaved miRNA and miRC target categories found with degradome analyses. Figure S3. GO terms of targets of known miRNAs and miRCs identified in *A. donax*. Figure S4. 5′ RACE on transcript TR4471, coding for a 32 kDa dirigent-like protein targeted by miR156d-3p.
