# Supplementary material for: Identification of Known and Novel Arundo donax L. MicroRNAs and Their Targets Using High-Throughput Sequencing and Degradome Analysis
Source: Life (Basel). 2022 Apr 27;12(5):651. doi: 10.3390/life12050651 (PMC9142972; doi:10.3390/life12050651)
Supplement: Supplementary file 1 [file life-12-00651-s001.zip › FigureS4.pdf]

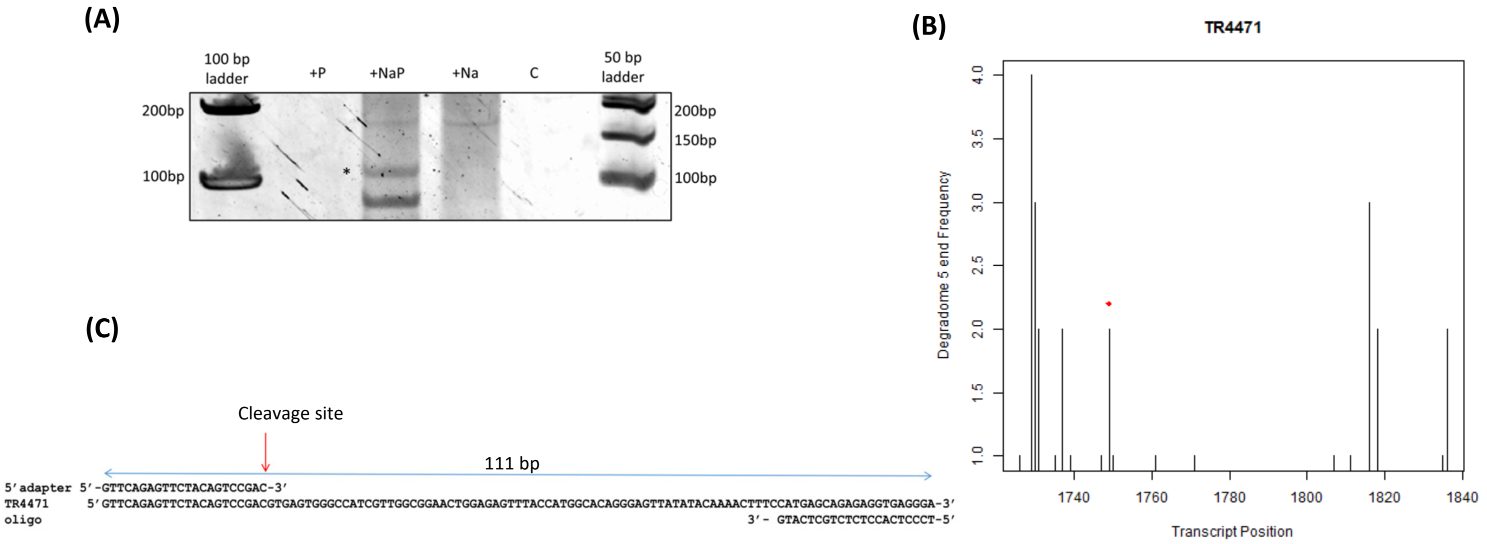


**Figure S4** - 5' RACE on transcript TR4471, coding for a 32kDa dirigent-like protein targeted by miR156d-3p . A) Fragment of 110 bp amplified by 5'RACE only in +NaP treatment. (*) indicates the eluted band; B) CleaveLand plot for TR4471 in the range of 1700-1840 nt, the red dot indicates the slicing site ; C) Sequence of TR4471 amplified by 5'RACE, with indication of 5' adapter and specific oligo alignment, the red arrow indicates the predicted cleavage site and blue arrow indicates the expected fragment length.
