# Supplementary material for: Identification of Known and Novel Arundo donax L. MicroRNAs and Their Targets Using High-Throughput Sequencing and Degradome Analysis
Source: Life (Basel). 2022 Apr 27;12(5):651. doi: 10.3390/life12050651 (PMC9142972; doi:10.3390/life12050651)
Supplement: Supplementary file 1 [file life-12-00651-s001.zip › TableS1.pdf]

Table S1 - primer sequences and melting temperature used for PCRs in 5’ RACE

Name	Sequence	Melting temperature
5’ adapter_fw	5’-GTTCAGAGTTCTACAGTCCGAC-3’	47°C
TR10651_rev	5’-CCTTGAACCACCTCTTGTCGC-3’	56°C
TR4471_rev	5’-TCCCTCACCTCTCTGCTCATG-3’	54°C
