# Supplementary material for: Identification of Known and Novel Arundo donax L. MicroRNAs and Their Targets Using High-Throughput Sequencing and Degradome Analysis
Source: Life (Basel). 2022 Apr 27;12(5):651. doi: 10.3390/life12050651 (PMC9142972; doi:10.3390/life12050651)
Supplement: Supplementary file 1 [file life-12-00651-s001.zip › TableS2.pdf]

Table S2 - Reads statistics of small RNAs high-throughput sequencing

**Treatment**	**raw reads (redundant)**	**raw reads (non-redundant)**	**clean reads (redundant)**	**clean reads (non-redundant)**
C	12,616,870	2,008,550	2,766,570	1,105,041
+P	14,180,274	2,751,532	4,096,100	1,704,026
+Na	11,403,618	1,963,790	2,545,750	983,071
+NaP	9,813,447	1,741,065	2,683,450	880,357
