# Supplementary figures and images for: Identification of Known and Novel Arundo donax L. MicroRNAs and Their Targets Using High-Throughput Sequencing and Degradome Analysis
Source: Life (Basel). 2022 Apr 27;12(5):651. doi: 10.3390/life12050651 (PMC9142972; doi:10.3390/life12050651)

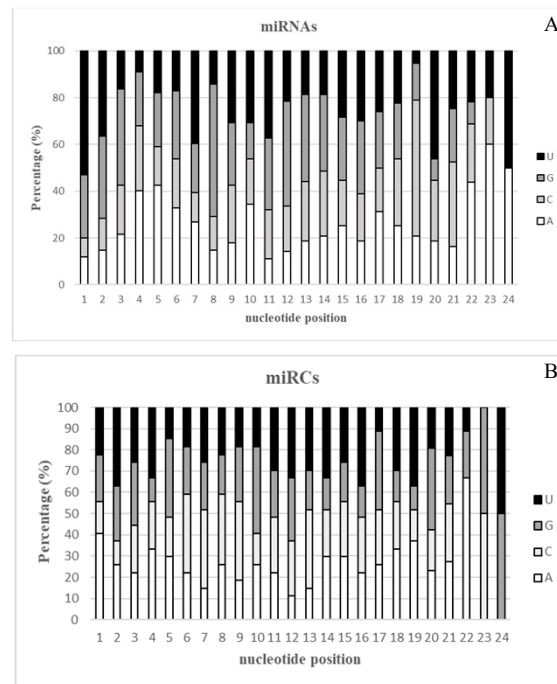

**Figure S1** - Nucleotide composition of miRNAs (A) and miRCs (B) found in *A. donax*

Supplement: Supplementary file 1 [file life-12-00651-s001.zip › FigureS1.pdf]

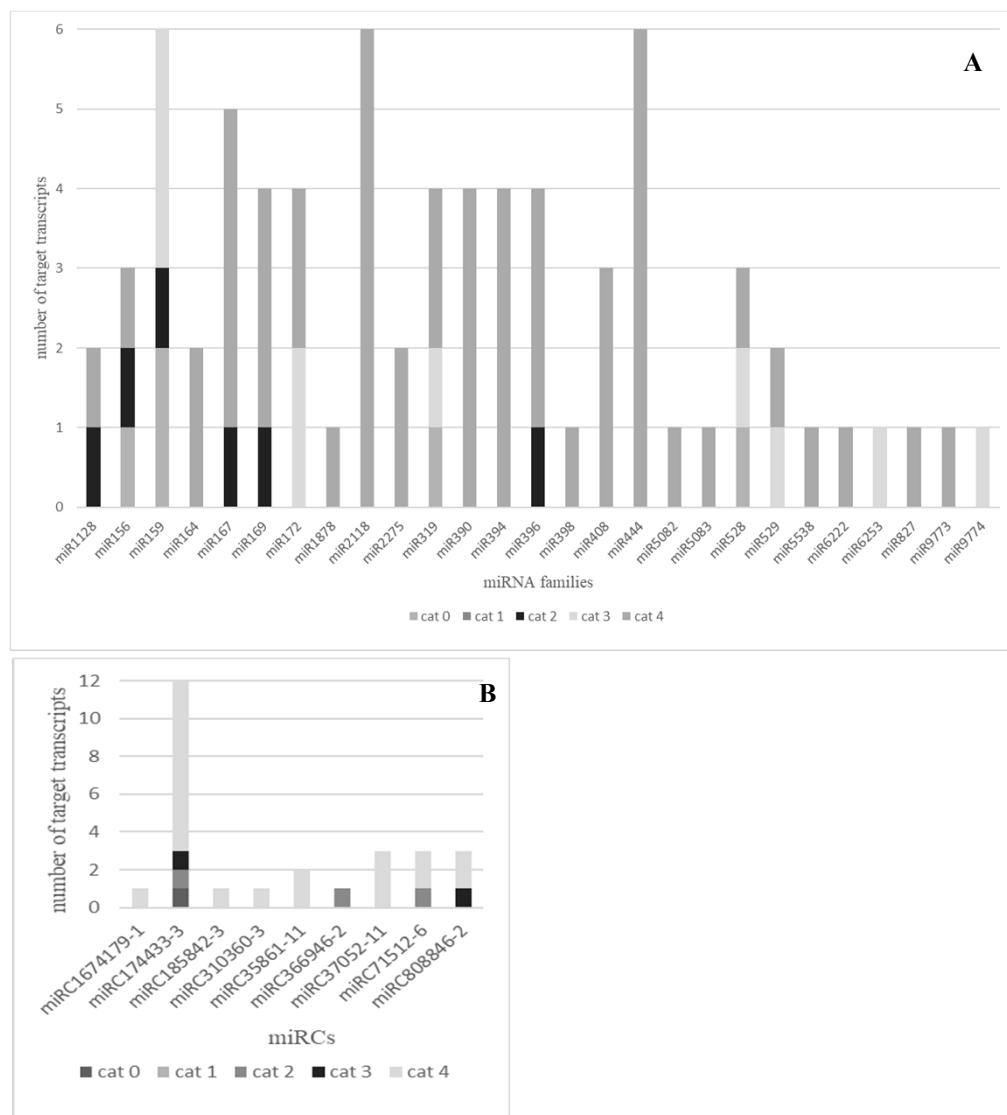

**Figure S2** - Summary of cleaved miRNA and miRC target categories found with degradome analyses

Supplement: Supplementary file 1 [file life-12-00651-s001.zip › FigureS2.pdf]

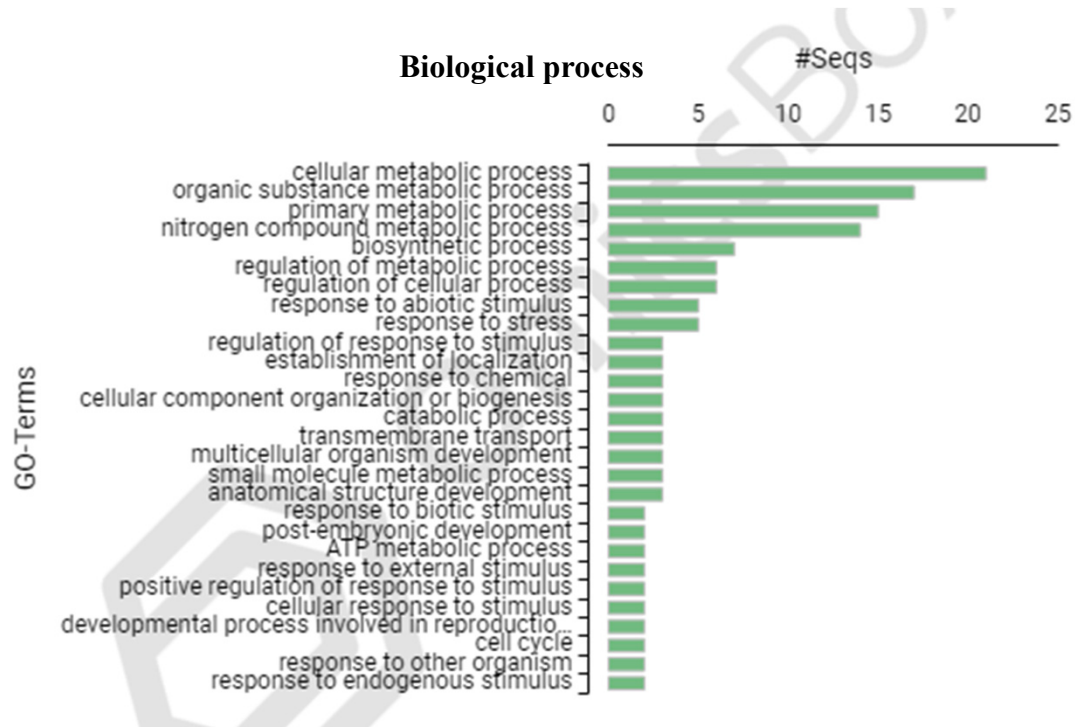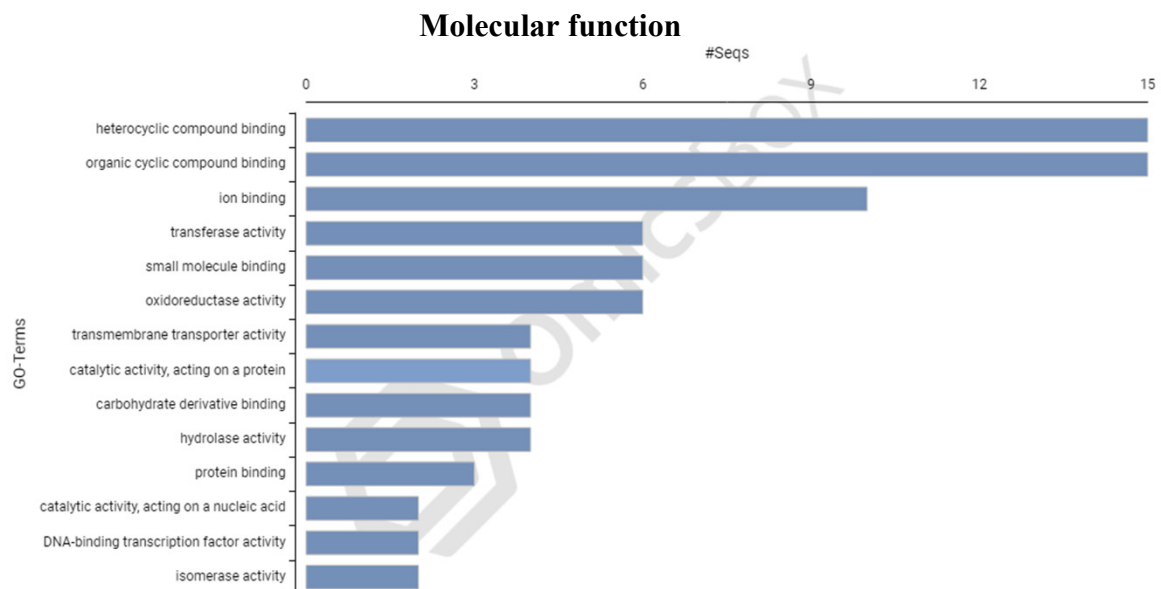

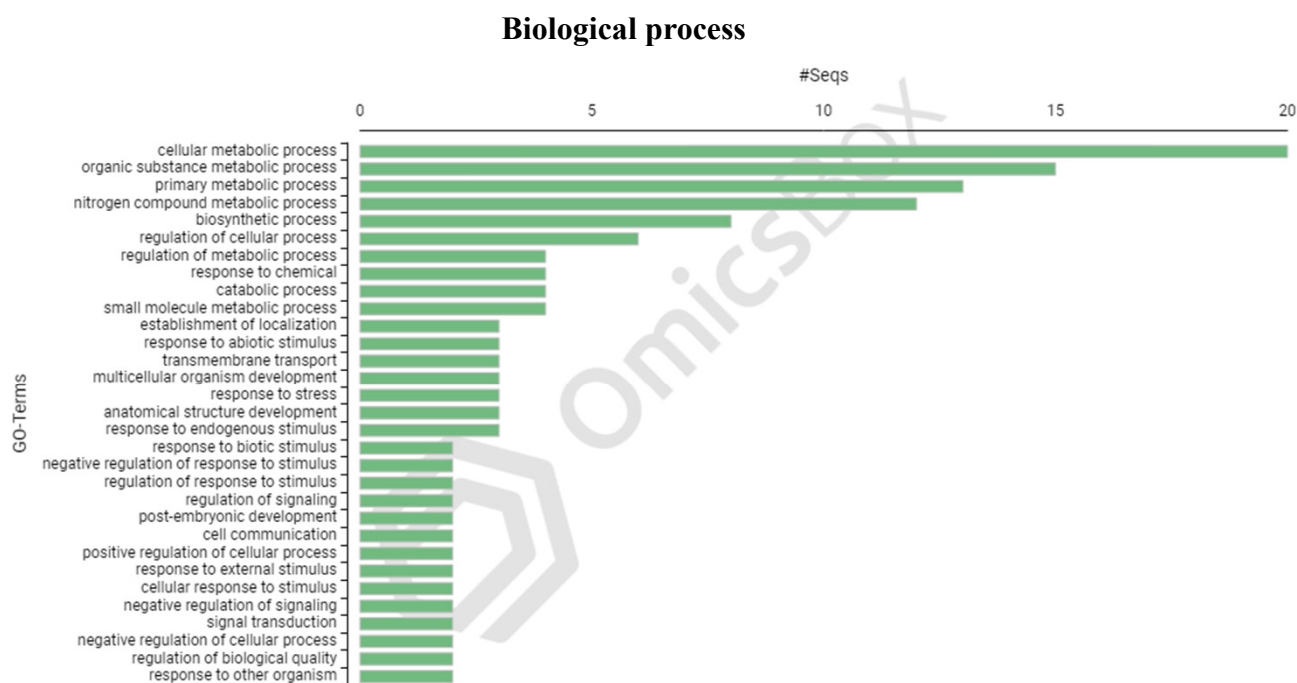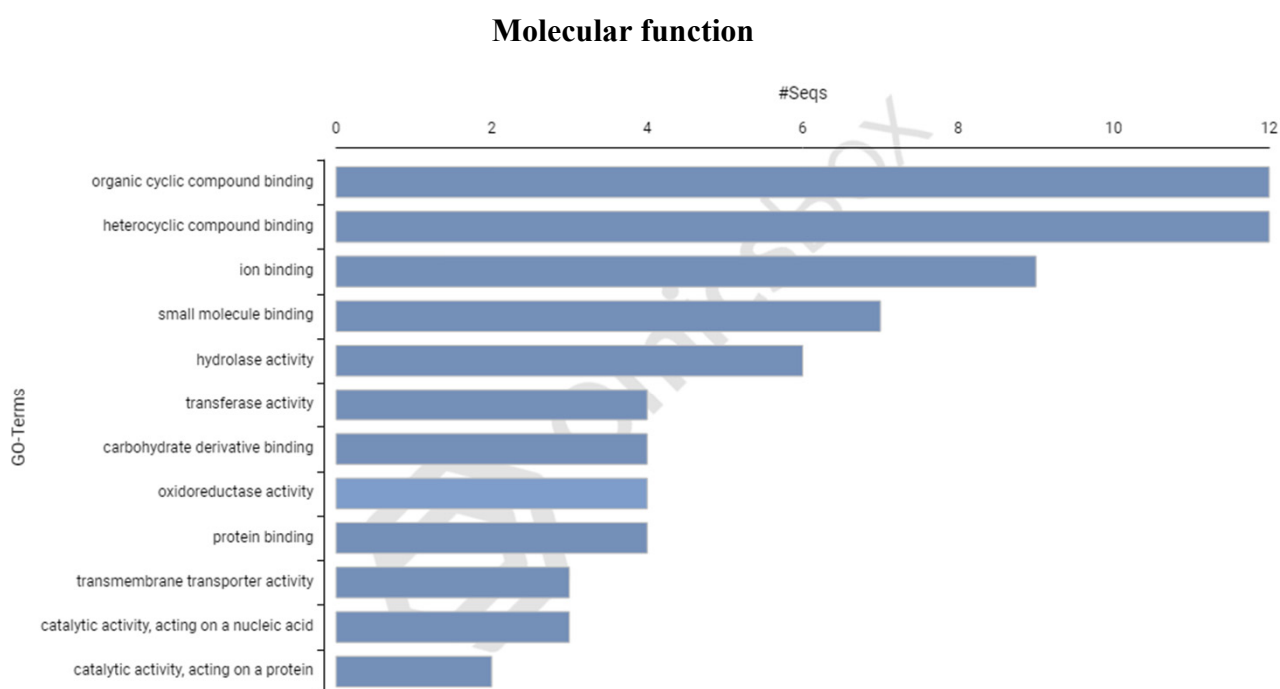

**Figure S3** - GO terms of targets of known miRNAs (A and B) and miRCs (C and D) identified in *A. donax*

Supplement: Supplementary file 1 [file life-12-00651-s001.zip › FigureS3.pdf]
